# The Effectiveness of Dance Interventions on Health-Related Outcomes in Perimenopausal, Menopausal, and Postmenopausal Women: A Systematic Review and Meta-Analysis

**DOI:** 10.3390/healthcare13080881

**Published:** 2025-04-11

**Authors:** Diying Liao, Lili Mo, Maowei Chen

**Affiliations:** 1 Graduate School of Global Convergence, Kangwon National University, Chuncheon 24341, Republic of Korea; ldy9074532748@163.com (D.L.); m17307149375@163.com (L.M.); 2 Department of Global Convergence, Kangwon National University, Chuncheon 24341, Republic of Korea

**Keywords:** dance intervention, health-related outcomes, meta-analysis, menopausal symptoms, systematic review, women

## Abstract

**Background/Objectives**: Dance intervention, as a non-pharmacological therapy, has shown promising potential in alleviating menopausal symptoms among perimenopausal, menopausal, and postmenopausal women. However, a systematic evaluation of its overall effectiveness based on existing trials remains unavailable. This study aims to investigate the effectiveness of dance intervention on health-related outcomes in perimenopausal, menopausal, and postmenopausal women through a systematic review and meta-analysis. **Methods**: This study systematically searched the relevant databases on 18 October 2024. The risk of bias was assessed using the Cochrane RoB 2 and ROBINS-I tools. Meta-analysis was performed using Review Manager version 5.4. software. For results unsuitable for meta-analysis, narrative synthesis was conducted. The study was registered in PROSPERO (number: CRD42024613134). **Results**: Meta-analysis demonstrated significant positive effects of dance intervention on psychological symptoms, including depression (I^2^ = 87%, *p* < 0.001), anxiety (I^2^ = 90%, *p* = 0.01), vitality (I^2^ = 0%, *p* = 0.03), interpersonal relationships (I^2^ = 0%, *p* < 0.001), and somatization (I^2^ = 85%, *p* = 0.01), in menopausal women, but no significant impact was observed on psychotic symptoms (I^2^ = 89%, *p* = 0.33). However, the high heterogeneity suggests the presence of potential confounding factors among studies. Sensitivity analysis indicated that the flexibility of the intervention protocol and intra-group differences among participants may have been the main sources of heterogeneity. Further subgroup analysis revealed that interventions conducted less than three times per week had significant effects on depressive symptoms (SMD = −1.93), while a total intervention duration of ≤1800 min significantly improved anxiety symptoms (SMD = −2.15). **Conclusions**: Dance interventions have significant positive effects on health-related outcomes in perimenopausal, menopausal, and postmenopausal women, except for psychotic symptoms, offering a promising intervention option for clinical practice.

## 1. Introduction

Menopause is defined as the permanent cessation of menses resulting from reduced ovarian hormone secretion that occurs naturally or is induced by surgery, chemotherapy, or radiation. Natural menopause is recognized after 12 months of amenorrhea that is not associated with a pathologic cause [1]. Menopause typically occurs between the ages of 40 and 50, though the average age of onset varies across countries and regions. In the United States, the average age of menopause is 51 years, while in China, it is 46.2 years [2]. In Korea, the average age ranges between 50 and 52 years [3]. Menopausal symptoms commonly emerge during three distinct phases: perimenopause, menopause, and postmenopause. Perimenopause refers to the transitional stage when menstruation has not ceased entirely, but becomes irregular. Menopause is defined as the point of the last natural menstrual period. Postmenopause begins 12 months after the final menstrual period, and continues as a prolonged phase of amenorrhea [4].

During these phases, women may experience significant physiological and psychological changes, often manifesting as symptoms such as hot flashes, night sweats, mood swings, heart palpitations, headaches, and sleep disturbances [5]. In severe cases, these symptoms can negatively impact health, well-being, and daily life, leading to a substantial reduction in quality of life. The impact of these symptoms on overall health is a critical focus of menopause research and interventions.

The treatment of menopausal symptoms includes pharmacological and non-pharmacological (physical) approaches. Pharmacological treatments comprise hormone therapy (HT) and non-hormonal drug therapy (NHDT). While HT has been shown to alleviate menopausal symptoms effectively, it is associated with increased risks, such as a higher likelihood of breast cancer, stroke, and thromboembolic events [6]. NHDT, on the other hand, not only alleviates menopausal symptoms, but also reduces the risk of osteoporosis and coronary heart disease (CHD). However, it may also lead to cardiovascular complications and breast cancer as side effects [7].

In contrast, non-pharmacological treatments are generally preferred by perimenopausal, menopausal, and postmenopausal women due to their safety and acceptability [8]. Dance intervention, as a form of non-pharmacological therapy, has been demonstrated to effectively improve physiological and psychological symptoms. Recent reviews of dance intervention highlight its positive effects on walking/gait, balance/postural control, and range of motion in patients with cerebral palsy [9]. Another study [10] reported that dance intervention significantly improved body image, self-esteem, physical function, grip strength, life satisfaction, and psychological components of quality of life in cancer patients, while also showing notable positive effects on depression and fatigue. Dance interventions can also improve negative symptoms and social impairments in patients with autism [11], and are also used as a complementary tool for the rehabilitation of stroke survivors in the subacute and chronic stages [12].

In recent years, dance intervention has increasingly been applied to perimenopausal, menopausal, and postmenopausal women, with studies suggesting its potential benefits for alleviating menopause-related symptoms. For instance, Rossmeissl et al. [13] evaluated the effects of Zumba dance intervention in postmenopausal women, finding significant improvements in quality of life and reductions in menopausal symptoms. However, no substantial impact on cardiopulmonary health was observed. Similarly, a study by Yang et al. [14] demonstrated that line dancing significantly alleviated depression, anxiety, paranoia, and sleep disturbances in menopausal women, enhancing psychological well-being and overall quality of life.

Although existing studies have preliminarily validated the feasibility of dance interventions as a non-pharmacological treatment for menopausal symptoms, most evidence is derived from independent experiments with small sample sizes and methodological variations, resulting in inconsistencies in effect evaluation and measurement outcomes. This limitation underscores the need for systematic reviews and meta-analyses of multiple experimental results. Only by integrating existing data can the overall effect of dance interventions on health-related outcomes in perimenopausal, menopausal, and postmenopausal women be quantitatively assessed, and further insights into the underlying mechanisms and inter-study heterogeneity be obtained. Addressing this knowledge gap not only helps to reveal the shortcomings of current research, but also provides a more robust evidence base for clinical application and dissemination, ultimately advancing the development of more effective non-pharmacological treatment strategies.

This study systematically reviews and analyzes existing research to determine the effectiveness of dance interventions on menopausal health outcomes and identify areas for further investigation. The findings are expected to provide evidence-based guidance for clinicians and researchers, facilitating the development of effective dance intervention strategies to improve the health and well-being of perimenopausal, menopausal, and postmenopausal women.

## 2. Materials and Methods

This systematic review and meta-analysis protocol was registered and published in PROSPERO (Registration Number: CRD42024613134). During the course of the study, appropriate revisions were made to the registered protocol based on research needs and practical considerations, which were approved by PROSPERO.

### 2.1. Source Search Strategy

To evaluate the effectiveness of dance interventions on health-related outcomes in perimenopausal, menopausal, and postmenopausal women, a systematic search was conducted across multiple databases, including Cochrane, PubMed, Embase, Web of Science, Scopus, RISS, and China National Knowledge Infrastructure (CNKI). The search covered the period from the inception of each database to 18 October 2024, with no language restrictions.

For the Cochrane Central Register of Controlled Trials, the search terms used for menopause included the following: (“Menopause” [MeSH] OR “Menopausal Transition” [MeSH] OR “Premature Menopause” [MeSH] OR “Perimenopause” [MeSH] OR “Postmenopause” [MeSH] OR “Pre-Menopause” OR “Pre-menopausal Period” OR “Perimenopausal Period” OR “Post-Menopausal Period” OR “Postmenopausal Period” OR “Post-Menopauses”).

For dance interventions, the search terms included the following: (“Dance Therapy” [MeSH] OR “Dancing” OR “Dance” OR “Ballet” OR “Jazz Dance” OR “Tap Dance” OR “Modern Dance” OR “Hip-Hop Dance” OR “Line Dancing” OR “Salsa Dancing” OR “Square Dance”).

The two groups of search terms were combined using the “AND” operator. Minor adjustments to the search terms were made for other databases to ensure comprehensive retrieval of relevant studies.

### 2.2. Research Options

One research team member conducted an initial database search to identify potentially relevant studies, and cross-checked the search terms with another team member. The retrieved articles and their titles were imported into EndNote for screening. After removing duplicates in EndNote 21, two independent reviewers evaluated the studies based on predefined inclusion and exclusion criteria.

The titles/abstracts and full texts were independently screened by the two reviewers. Any disagreements were resolved through discussion and mutual explanation of viewpoints until a consensus was reached. If consensus could not be achieved, a third reviewer was consulted for arbitration.

### 2.3. Inclusion and Exclusion Criteria

#### 2.3.1. Inclusion Criteria

Type of Participants: Women who were perimenopausal, menopausal, or postmenopausal.

Intervention Group: Dance interventions of any type, without restrictions on the style, duration, content, frequency, or setting of the intervention.

Control Group: Any control group other than dance interventions, including usual care, pharmacological treatments, mental health interventions, or exercise groups.

Outcome Measures: Depression scales, anxiety scales, menopause rating scales, and any other reported outcomes to assess changes in physical function, activity levels, or psychological status among perimenopausal, menopausal, or postmenopausal women participating in dance interventions.

Type of study: Controlled trial.

#### 2.3.2. Exclusion Criteria

Type of Participants: Menopausal women with a history of cancer, heart disease, thyroid disease, or related psychiatric disorders.

Intervention Group: Invasive physical therapy methods, such as acupuncture, women undergoing menopause due to surgical ovarian removal, or those using contraceptives.

Control Group: Studies without a control group.

Outcome Measures: Studies that did not report any outcome measurement data.

Type of study: Single-arm studies.

### 2.4. Data Extraction

A customized data extraction tool was designed in Excel (Microsoft Inc., Redmond, WA, USA) to extract data. Two researchers independently extracted the data and cross-verified them to ensure accuracy and completeness. Discrepancies were resolved through arbitration by a third researcher. Extracted data included study authors, population source, sample size, participant age, intervention details (type of dance, protocol, number of sessions, duration), outcome measures, and results (mean values and standard deviations). Data were entered and double-checked in the tool to minimize human error and enhance the reliability of the research.

### 2.5. Quality Assessment

Two independent authors rigorously assessed the quality of all included studies. For randomized controlled trials (RCTs), the Cochrane Risk of Bias Tool (ROB2.0) [15] was employed to evaluate the overall risk of bias based on ratings in domains such as the randomization process, deviations from intended interventions, missing outcome data, outcome measurement, and selective reporting. Studies were classified as “low risk” if all key domains were rated as low-risk; as “moderate risk” if at least one domain was rated as moderate-risk, with no key domain rated as high-risk; and as “high risk” if at least one key domain was rated as high-risk, or multiple non-key domains exhibited moderate or high risk.

For non-randomized intervention studies, the Risk of Bias in Non-Randomized Studies of Interventions (ROBINS-I) tool [16] was used, covering domains including confounding, selection bias, classification bias, missing data, measurement bias, and reporting bias. The overall risk of bias was determined as follows: studies were classified as “low risk” if all domains were rated as low-risk; as “moderate risk” if at least one domain was rated as moderate-risk, and none were rated as high-risk; and as “high risk” if at least one domain was rated as high-risk.

If consensus could not be reached, a third author intervened to make the final decision on the risk of bias rating. Subsequently, the research team graded the overall quality of the included studies (low-, moderate-, or high-risk) based on the risk of bias assessment, and explored the potential impact of bias on the interpretation of the study findings. This stratified evaluation framework not only ensured objectivity and rigor in quality assessment, but also enhanced the reliability and persuasiveness of the study outcomes.

### 2.6. Data Synthesis and Analysis

Statistical analyses were conducted using Review Manager version 5.4. Standardized Mean Differences (SMDs) or Mean Differences (MDs) were calculated for the included studies. When measurement tools differed among studies, SMD was used to standardize the data for comparability. When measurement tools were consistent, MD was directly applied. For pooled analyses, the random-effects model (I^2^ ≥ 50%) or fixed-effects model (I^2^ < 50%) was used based on the degree of heterogeneity, assessed via the I² statistic; for heterogeneity exceeding 50%, subgroup analyses were performed to explore sources of variability.

## 3. Results

### 3.1. Search Results

**Figure 1:**
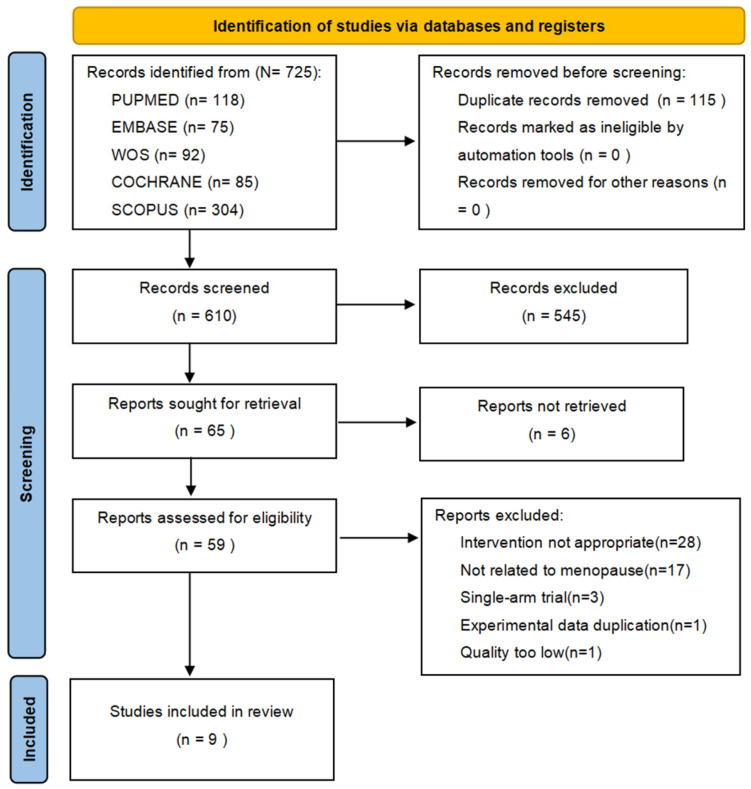
PRISMA (2020) diagram of study screening and selection.

The search yielded 725 articles, of which 115 duplicate records were removed. After screening titles and abstracts, 545 articles were excluded, and six were inaccessible. A total of 59 studies underwent full-text evaluation, and nine studies were ultimately included. Figure 1 presents the flowchart of the database search.

### 3.2. Description of Included Studies

The nine studies [17,18,19,20,21,22,23,24,25,26] included controlled trials published between 2004 and 2024, and encompassed studies from multiple countries. Four studies were conducted in Korea [17,18,19,20], two in China [21,22], and the remaining three in Tunisia [23], Brazil [24], and Thailand [25], respectively, with one [20] being a pilot study. These studies involved a total of 435 participants, with 219 in the intervention group (50.3%) and 216 in the control group (49.7%). Participants’ ages ranged from 40 to 60 years. The study types included seven randomized controlled trials and two non-randomized controlled trials.

Regarding the selection of participants, to ensure uniformity of the experimental population, Waer et al. [23] selected women aged 55–60 with mild menopause; Martins [24] selected menopausal women aged 40–59; two studies [21,22] selected women with menstrual disorders between the ages of 45 and 50; Pan [21] selected women over 40 years old whose menstruation had been suspended for at least 6 consecutive months; So et al. [17] selected women who had been in menopause for less than one year; Kim [19] had a wider population selection, and included menopausal women aged 40–60; and in Kim [20], middle-aged menopausal women who had been in menopause for less than 5 years were selected.

Although the studies involved interdisciplinary research teams, only four [17,23,24,25,26] had interventions conducted by professionals with teaching experience. Two studies [21,22] employed interventionists who were members of the research team, while the remaining three [18,19,20] did not specify the background of the intervention implementers.

The frequency of interventions ranged from two to six sessions per week, with the total intervention duration varying between 1080 min [25] and 7200 min [22]. Various dance forms were employed, including Zumba [22], jazz [23], traditional dance [20,26], sports dance [18,19,21,22], and dance movement therapy (DMT) [17].

In terms of measurement results, a total of four studies [17,19,23,24] evaluated the mental health of perimenopausal, menopausal, and postmenopausal women, focusing on depression, anxiety, vitality, mental state, etc. These studies assessed the impact of dance intervention on the participants’ mental health through psychological scales, questionnaires, and other tools; in addition, six studies [18,19,20,22,23,26] focused on evaluating the physiological indicators of menopausal women. These studies examined the effects of dance intervention on the physiological health of perimenopausal, menopausal, and postmenopausal women. The indicators measured included lung function, muscle mass, fat mass, body shape (such as measurements, waist-to-hip ratio, etc.), estrogen levels, blood lipid levels, etc. The monitoring of these physiological parameters helps to gain an understanding of the regulatory effect of dance on overall physical function; two studies [23,24] paid special attention to the evaluation criteria for menopausal symptoms. Table 1 provides a detailed description of dance interventions’ specific content and protocols across the included studies.

### 3.3. Assessment of Risk of Bias

**Figure 2:**
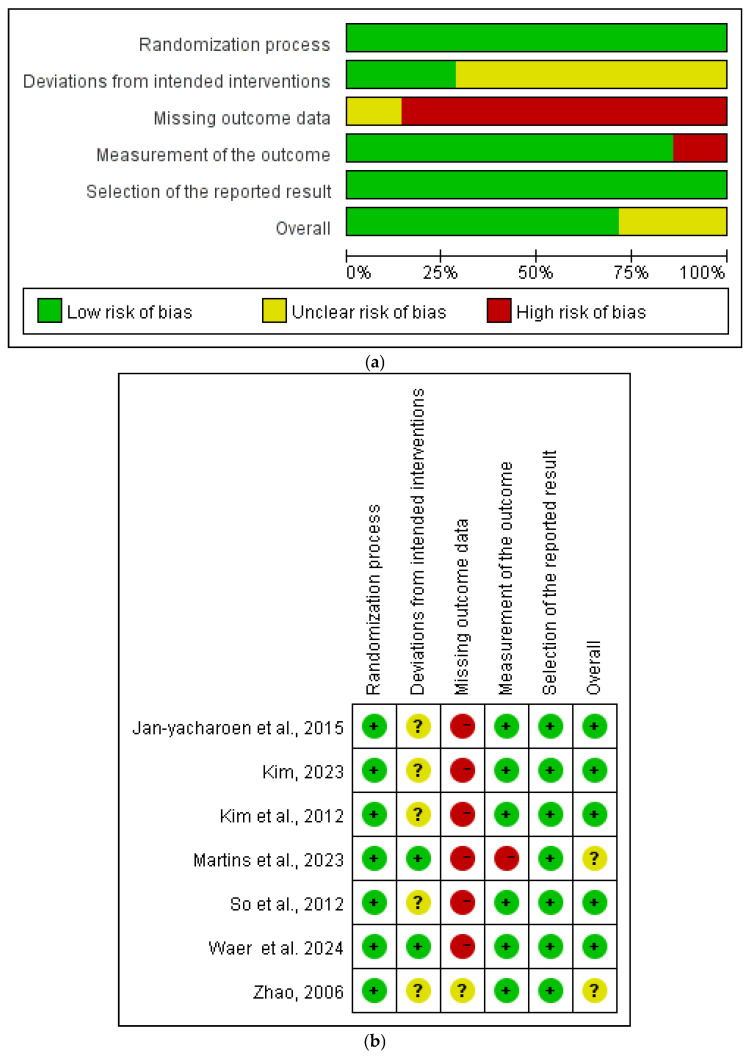
(**a**) Graphical map of RCT bias analysis. (**b**) Summary map of RCT bias analysis [17,18,20,22,23,24,25].

As shown in Figure 2a,b, based on Cochrane’s official recommendations, this study systematically evaluated the seven included randomized controlled trials (RCTs) using the Review Manager version 5.4 software in conjunction with the latest Risk of Bias assessment tool (ROB2.0). The evaluation comprehensively analyzed the risk of bias across five domains: the randomization process, intervention bias, missing data, outcome measurement, and selective reporting.

The results showed that one study [24] utilized online randomization software, while the remaining six studies explicitly reported random allocation, but did not clearly describe the details of the randomization process. Overall, the randomization process was rated as low-risk. Regarding intervention implementation, two studies [22,24] were rated as high-risk due to insufficient assessor blinding, while the remaining studies were marked as having an unclear risk due to insufficient details. For data completeness, one study [24] was rated as high-risk due to low participant adherence leading to data loss, while the other six studies fully reported outcome data, demonstrating high reliability. In the domains of outcome measurement and selective reporting bias, most studies were rated as low-risk, with only a few marked as having an unclear risk due to insufficient methodological descriptions. In summary, the risk of bias in the included studies was at a moderate level of credibility, indicating a certain degree of reliability in the results. However, there is room for improvement in areas such as the randomization process, implementation of blinding, and data completeness.

**Figure 3:**
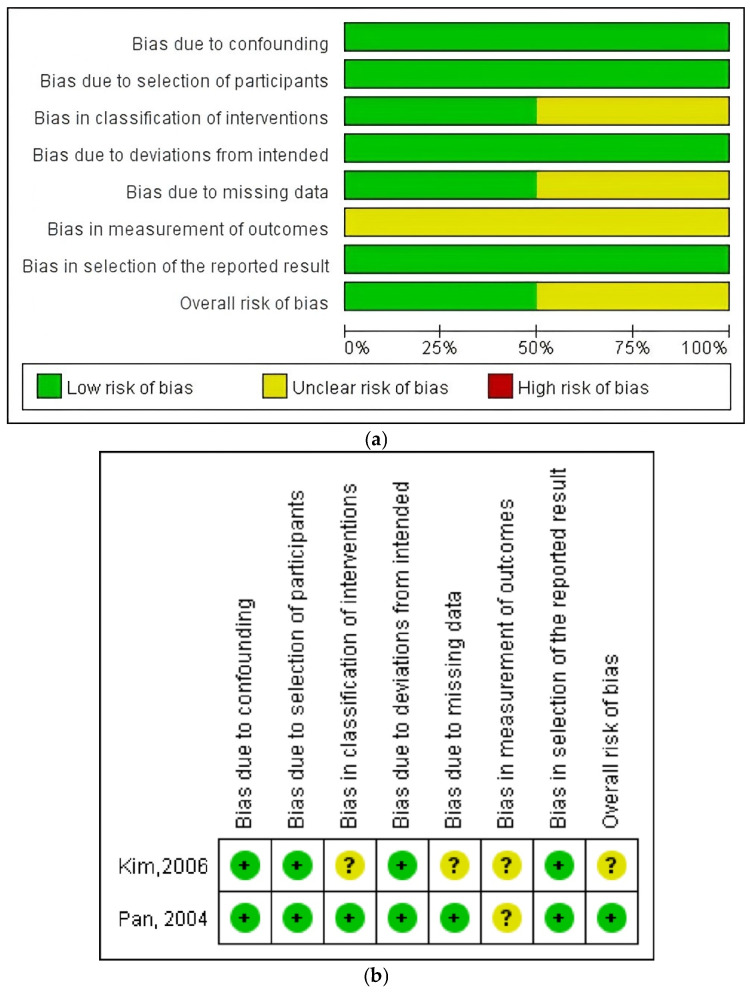
(**a**) Graphical map of non-RCT bias analysis. (**b**) Summary map of non-RCT bias analysis [19,21].

As shown in Figure 3a,b, the authors assessed the two included non-randomized studies [19,21] using the Risk of Bias in Non-Randomized Studies of Interventions tool (ROBINS-I). Both studies were rated as low-risk for confounding bias, participant selection bias, pre-specified intervention bias, and selective reporting bias, suggesting robust control of these sources of bias. However, in terms of intervention implementation, one study [19] did not classify the interventions, resulting in an unclear risk rating, while the other [21] was rated as low-risk. Additionally, neither study provided sufficient details regarding outcome measurement bias, which was consequently rated as posing an unclear risk for both studies. Overall, despite some unclear risk points, the two non-randomized studies demonstrated low risk in key domains such as confounding bias and selective reporting bias, indicating relatively high levels of credibility. These results suggest that the conclusions drawn from these studies are largely reliable.

### 3.4. Meta-Analysis Results

**Figure 4:**
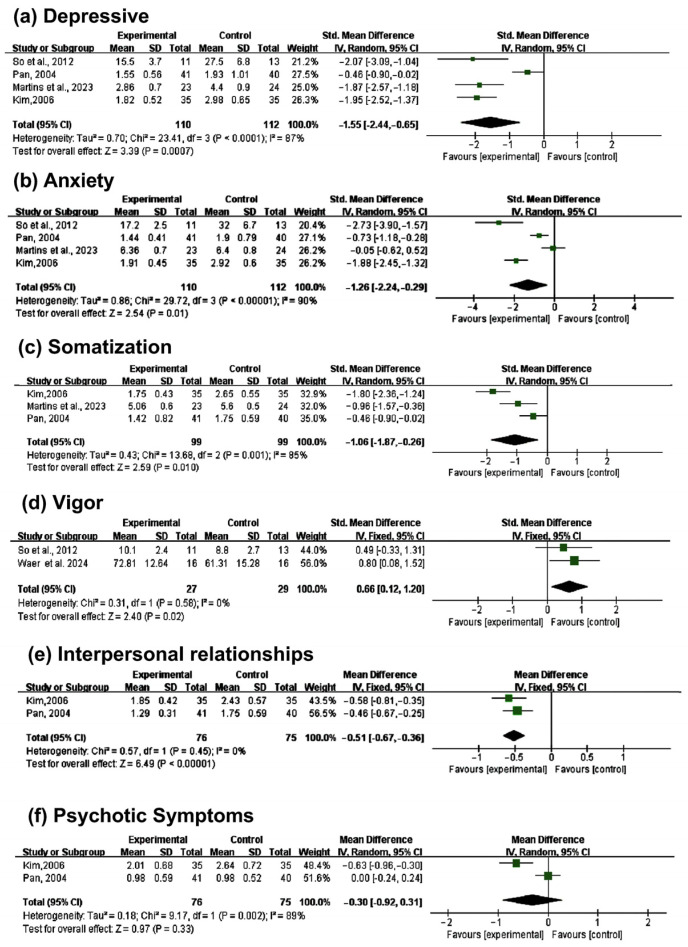
Forest plot for dance vs. control group [17,19,21,23,24].

This meta-analysis included five studies [17,19,21,23,24] involving 251 participants. Forest plots were generated for five psychological health-related outcomes: depression, anxiety, somatization, vitality, interpersonal relationships, and psychotic symptoms. Subgroup analyses were also conducted for depression and anxiety. The characteristics of the meta-analysis data are detailed in Figure 4.

#### 3.4.1. Depression

Four studies [17,19,21,24] assessed the impact of dance interventions on depression. The results showed high heterogeneity (I^2^ = 87%, *p* < 0.001), and a random-effects model was therefore used. The pooled effect size indicated that dance interventions significantly reduced depression scores compared to the control group (SMD = −1.55, 95% CI [−2.44, −0.65]; I^2^ = 87%, *p* < 0.001). Since the *p*-value was below 0.05, the difference was statistically significant compared to the control group. According to Cohen’s effect size criteria (with an SMD > 0.8 considered a large effect), an SMD of −1.55 demonstrates an extremely significant intervention effect that generally has important clinical implications in practice. However, the high heterogeneity indicates significant differences among the studies, which may affect the generalizability and external validity of the findings; therefore, these results should be interpreted with caution. The results for depression are shown in Figure 4a.

#### 3.4.2. Anxiety

At the same time, the four studies [17,19,21,24] that evaluated “depression” also focused on the impact of dance interventions on anxiety. The results showed high heterogeneity (I^2^ = 90%, *p* = 0.01), necessitating a random-effects model. The pooled effect size indicated that dance significantly reduced anxiety scores compared to the control group (SMD = −1.26, 95% CI [−2.24, −0.29]; I^2^ = 90%, *p* = 0.01), with the difference being statistically significant. According to Cohen’s effect size criteria (with an SMD > 0.8 considered a large effect), an SMD of −1.26 demonstrates an extremely significant intervention effect that generally has important clinical implications in practice. However, the high heterogeneity suggests significant differences among the studies, which may affect the generalizability and external validity of the results; therefore, these conclusions should be interpreted with caution. The results for anxiety are shown in Figure 4b.

#### 3.4.3. Somatization

Three studies [19,21,24] assessed the effects of dance on somatization. The results showed high heterogeneity (I^2^ = 85%, *p* = 0.010), prompting the use of a random-effects model. The pooled effect size revealed that dance significantly reduced somatization scores compared to the control group (SMD = −1.06, 95% CI [−1.87, −0.26]; I^2^ = 85%, *p* = 0.010), with a statistically significant difference. According to Cohen’s effect size criteria (with an SMD > 0.8 considered a large effect), an SMD of −1.06 demonstrates an extremely significant intervention effect that generally has important clinical implications in practice. However, the high heterogeneity suggests significant differences among the studies, which may affect the generalizability and external validity of the results; therefore, these conclusions should be interpreted with caution. The results for somatization are shown in Figure 4c.

#### 3.4.4. Vigor

Two studies [17,23] evaluated the impact of dance on vitality. The results showed low heterogeneity (I^2^ = 0%, *p* = 0.02), and a random-effects model was applied. The pooled effect size demonstrated that dance significantly improved vitality scores compared to the control group (SMD = −0.62, 95% CI [−1.18, −0.07]; I^2^ = 0%, *p* = 0.02), with a statistically significant difference. According to Cohen’s effect size criteria (with an SMD > 0.5 indicating a moderate effect), the observed SMD value of −0.62 in this study indicates that the intervention had a moderate effect. The results for vigor are shown in Figure 4d.

#### 3.4.5. Interpersonal Relationships

Two studies [19,21] assessed the effects of dance on interpersonal relationships. The results showed no heterogeneity (I^2^ = 0%, *p* < 0.001), and a fixed-effects model was used. The pooled effect size indicated that dance significantly improved interpersonal relationship scores in menopausal women compared to the control group (SMD = −0.51, 95% CI [−0.67, −0.36]; I^2^ = 0%, *p* < 0.001), with a statistically significant difference. According to Cohen’s effect size criteria (with an SMD > 0.5 indicating a moderate effect), the observed SMD value of −0.51 in this study indicates that the intervention had a moderate effect. The results for interpersonal relationships are shown in Figure 4e.

#### 3.4.6. Psychotic Symptoms

Two studies [19,21] evaluated the effects of dance on psychotic symptoms. The results showed high heterogeneity (I^2^ = 89%, *p* = 0.33), and a random-effects model was applied. The pooled effect size indicated that dance interventions did not significantly improve psychotic symptom scores in menopausal women compared to the control group (SMD = −0.30, 95% CI [−0.92, 0.31]; I^2^ = 89%, *p* = 0.33). Since the *p*-value was greater than 0.05, the difference was not statistically significant. According to Cohen’s effect size criteria (SMD > 0.2 indicates a small effect), the observed SMD value of −0.51 in this study indicates that the intervention had a small effect. However, the high heterogeneity indicates significant differences among the studies, which may affect the generalizability and external validity of the findings; therefore, these results should be interpreted with caution. The results for psychotic symptoms are shown in Figure 4f.

As shown in Figure 4, the heterogeneity of depression (I^2^ = 87%), anxiety (I^2^ = 90%), somatization (I^2^ = 85%), and psychotic symptoms (I^2^ = 89%) is exceptionally high, whereas the heterogeneity of vitality (I^2^ = 0%) and interpersonal relationships (I^2^ = 0%) is relatively low. Through sequential exclusion of the included studies, we found that the heterogeneity in depression was primarily driven by one study [21] (which employed a more flexible intervention protocol); the heterogeneity in anxiety was associated with two studies [24] (related to the total intervention duration); and the heterogeneity in somatization mainly stemmed from one study [19] (due to confounding factors within the participant group). Due to the inclusion of only two eligible studies, a comprehensive sensitivity analysis for psychotic symptoms could not be performed.

### 3.5. Publication Bias

Evidence suggests that, in general, the primary driving factor for controlled studies is the statistical significance of the research outcomes. Studies that fail to reach the perceived statistical significance threshold (*p* < 0.05) are less likely to be published [26].

**Figure 5:**
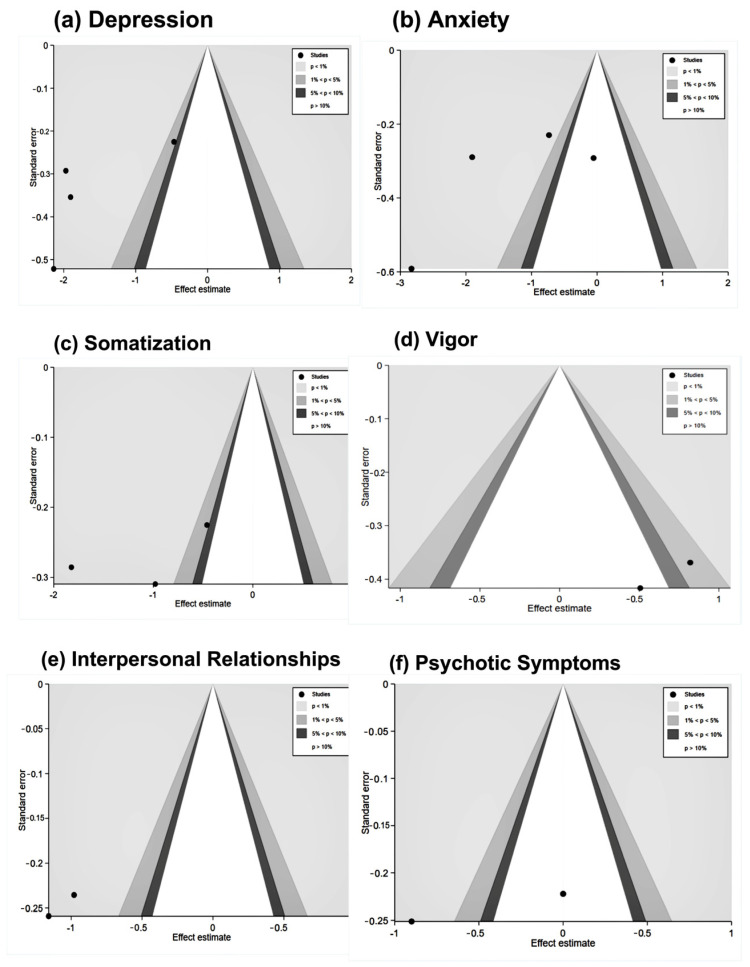
Contour-enhanced funnel plots of included studies [17,19,21,23,24].

To assess publication bias, the researchers performed Egger’s test [27]. They utilized contour-enhanced funnel plots to visualize publication bias and evaluate the robustness and reliability of the findings, as shown in Figure 5.

Egger’s test indicated high robustness for depression (*p* = 0.23), anxiety (*p* = 0.419) (although one study fell within the non-significant region, its proportion was small and did not substantially impact result robustness), and somatization (*p* = 0.51).

For vitality, interpersonal relationships, and psychotic symptoms, only two studies were available, preventing the execution of Egger’s test. However, contour-enhanced funnel plots revealed that vitality and psychotic symptoms each had one study falling within the non-significant region, suggesting the presence of some publication bias and lower robustness, warranting cautious interpretation.

In contrast, for interpersonal relationships, both studies were within the significant region, indicating no publication bias and high robustness.

### 3.6. Subgroup Analysis

**Figure 6:**
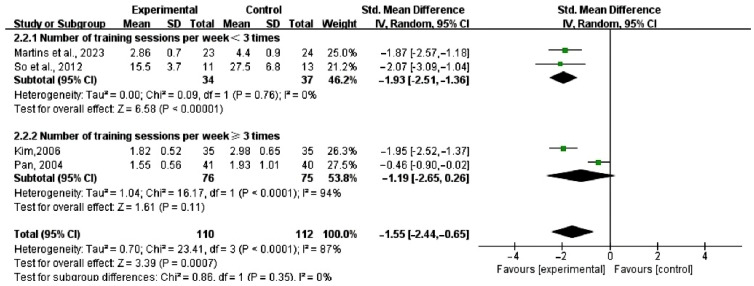
Subgroup analysis of impact of dance intervention on depressive symptoms [17,19,21,24].

**Figure 7:**
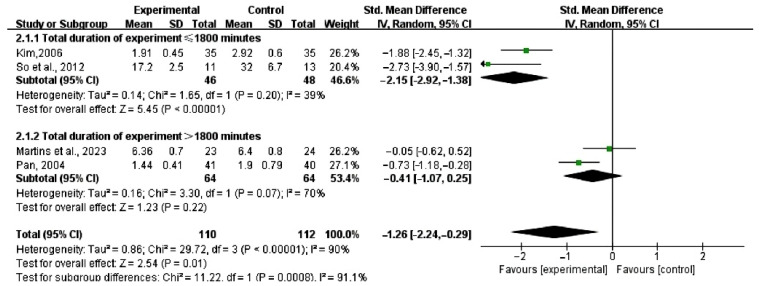
Subgroup analysis of impact of dance intervention on anxiety symptoms [17,19,21,24].

Due to the high heterogeneity observed in depression and anxiety outcomes, a subgroup analysis was conducted, as shown in Figure 6 and Figure 7.

Specifically, for depression (I^2^ > 50%), a subgroup analysis based on weekly intervention frequency (Figure 6) revealed that when dance interventions were performed less than three times per week (SMD = 1.93, 95% CI [2.51, 1.36]; I^2^ = 0%, *p* < 0.05), no heterogeneity was observed, and the difference was statistically significant; moreover, the contour-enhanced funnel plot showed that all studies fell within the significant region, indicating high robustness of the results. In contrast, for interventions conducted three or more times per week (SMD = 1.19, 95% CI [2.65, 0.26]; I^2^ = 94%, *p* > 0.05), severe heterogeneity was observed, and the difference was not statistically significant; additionally, one study in the contour-enhanced funnel plot, although its effect tended to favor the experimental group, was located near the border of the non-significant region, thereby limiting the overall robustness of the findings. These findings suggest that dance interventions performed fewer than three times per week are more effective in alleviating depressive symptoms in perimenopausal, menopausal, and postmenopausal women.

For anxiety (I^2^ > 50%), subgroup analyses were conducted based on total intervention duration (Figure 7). When the total duration was ≤1800 min (SMD = 2.15, 95% CI [2.92, 1.38]; I^2^ = 39%, *p* < 0.05), heterogeneity was low and the difference was statistically significant. Contour-enhanced funnel plot analysis also showed that all studies in this subgroup fell within the significant region, indicating robust results. However, when the total duration exceeded 1800 min (SMD = 0.41, 95% CI [1.07, 0.25]; I^2^ = 70%, *p* > 0.05), severe heterogeneity was observed, and the difference was not statistically significant; additionally, one study in the contour-enhanced funnel plot fell in the non-significant region, reducing the overall robustness of the findings. These results indicate that dance interventions with a total duration of ≤1800 min are more effective in alleviating anxiety symptoms in perimenopausal, menopausal, and postmenopausal women.

### 3.7. Narrative Results

Due to the variability in the design and objectives of the included studies, most results were not suitable for meta-analysis, and were instead synthesized through narrative review.

#### 3.7.1. Zumba Dance

Waer et al. [23] explored the effects of Zumba and Pilates training on functional ability, mood, and health-related quality of life in women. The findings indicated that both exercise modalities significantly improved walking speed, strength, dynamic balance, and functional activity capacity in menopausal women, along with emotional state improvements. However, Zumba demonstrated superior effects in areas such as social function (*p* < 0.001), mental health (*p* < 0.001), and role limitations due to emotional problems (*p* < 0.05), highlighting its unique advantages in enhancing psychological well-being.

#### 3.7.2. Jazz Dance

Martins et al. [24] conducted short-term (one month of intervention and post-intervention) and long-term (six-month follow-up) evaluations of the effects of jazz dance on menopausal symptoms (including physical, psychological, and urogenital domains) and specific psychological aspects (e.g., anxiety, depression, mood, stress, and perspectives on aging). The results revealed significant improvements in menopausal symptoms (*p* = 0.001), psychological domains (e.g., stress, *p* = 0.030), the integrity domain of the aging perspective (*p* = 0.011), and emotional confusion (*p* = 0.028). After one month of intervention, the intervention group exhibited significant improvements in the happiness domain (*p* = 0.043). Jazz dance demonstrated greater effectiveness in treating menopausal symptoms, depression, anxiety, and stress when the intervention lasted at least 16 weeks, confirming its positive impact on alleviating menopausal symptoms and enhancing psychological well-being.

#### 3.7.3. Sports Dance

Research on Sports Dance also revealed its beneficial effects on both the psychological and physiological health of menopausal women. Kim [19] reported reductions in anxiety, interpersonal difficulties, somatization, psychotic symptoms, and depressive mood in the intervention group compared to the control group. Pan [21] found that menopausal women who participated in sports dance for over one year experienced significantly reduced physical discomfort (e.g., menstrual irregularities, headaches, fatigue) and improved psychological health (e.g., reduced obsessive–compulsive symptoms, hostility, and phobia) compared to the control group. Physiologically, significant improvements were observed in body measurements (e.g., waist-to-hip ratio), lower heart rate, and enhanced lung capacity. Zhao [22] demonstrated that after sports dance interventions, the intervention group showed significant reductions in estradiol, progesterone, triglycerides, total cholesterol, and low-density lipoprotein cholesterol, alongside increases in high-density lipoprotein cholesterol (*p* < 0.05). The study suggested that moderate-intensity sports dance can effectively stimulate estrogen levels, improve lipid profiles, and help prevent chronic diseases in menopausal women. Kim [19] assessed physiological changes, and found significant improvements in bone density (*p* < 0.05), rectus femoris thickness (*p* < 0.05), muscle mass (*p* < 0.001), and fat mass (*p* < 0.001) in the intervention group.

#### 3.7.4. Traditional Dance

Janyacharoen et al. [25] evaluated the effects of traditional Thai dance on cardiopulmonary health in menopausal women. The intervention group showed significant improvements in the 6 min walk test (6MWT), pulmonary function tests, and thoracic expansion, while no changes were observed in the control group. Kim [20] assessed the effects of Korean traditional dance, and found significant post-intervention changes in immunoglobulin levels (IgA, *p* < 0.01; IgG, *p* < 0.01; IgM, *p* < 0.05) and body temperature in the intervention group, with no notable changes in the control group. The study suggested that Korean traditional dance could be a viable method for enhancing immune function.

#### 3.7.5. Dance Movement Therapy (DMT)

So et al. [17] examined the effects of DMT on anxiety, depression, and quality of life in menopausal women. The results showed significant improvements in physical and emotional health, capacity, and stability post-intervention (*p* < 0.05), indicating that DMT enhances physical and emotional well-being and improves quality of life.

## 4. Discussion

### 4.1. Summary of Findings

Research on dance interventions for perimenopausal, menopausal, and postmenopausal women has primarily focused on interventional experiments. This study systematically reviewed and conducted a meta-analysis of nine trials to assess the effectiveness of dance interventions on health-related outcomes in these populations. Four studies evaluated psychological outcomes, while six studies focused on physiological health-related results. The findings are discussed below by outcome category.

#### 4.1.1. Psychological Outcomes

The primary results of the meta-analysis indicate that dance interventions have a significantly positive effect on alleviating depression and anxiety, enhancing vitality, and improving interpersonal relationships and somatic symptoms in menopausal women. These findings are consistent with previous studies [28,29,30,31,32], all of which reported significant improvements in somatization, interpersonal difficulties, anxiety, depression, and vitality through dance interventions, thereby further validating the efficacy of dance as a psychological intervention for perimenopausal, menopausal, and postmenopausal women. However, no significant effect was found for psychotic symptoms.

Notably, the outcomes for vitality and interpersonal relationships exhibited low heterogeneity (I^2^ = 0%, *p* < 0.05). In contrast, depression (I^2^ = 87%, *p* < 0.00007), anxiety (I^2^ = 90%, *p* = 0.01), and somatization (I^2^ = 85%, *p* = 0.010) displayed high heterogeneity, suggesting substantial differences in intervention effects across studies. Subsequent sensitivity analyses revealed that the heterogeneity in depression was partly attributable to a study [21] that employed a relatively flexible intervention protocol, while the heterogeneity in anxiety was associated with confounding factors within the participant group in another study [19].

Regarding the efficacy of dance interventions, this study suggests that the effect may be influenced by factors such as intervention frequency and total duration. Based on this hypothesis, two subgroup analyses were conducted. The analysis revealed that groups with less than three interventions per week showed a significant effect in alleviating depressive symptoms (SMD = −1.93). This effect size markedly exceeded the clinical significance threshold of 0.8 [33], indicating a substantial and clinically meaningful difference in depression reduction between the intervention and control groups. Similarly, groups with a total intervention duration of ≤1800 min exhibited a significant improvement in anxiety symptoms (SMD = −2.15), with an effect size that also far surpassed the clinical significance threshold of 0.8, suggesting that dance interventions have a clinically meaningful effect in alleviating anxiety symptoms. These findings indicate that intervention designs may need to be optimized in terms of both frequency and total duration to enhance their effectiveness.

Nevertheless, given the relatively small sample sizes, these conclusions require further validation through larger-scale studies to determine the optimal intervention strategy and to mitigate the impact of heterogeneity.

#### 4.1.2. Physiological Outcomes

Studies have shown that, compared to premenopausal women, postmenopausal women exhibit a reduced ability to generate rapid propulsive force during the gait initiation phase [34]. Medium-to-high-intensity Zumba interventions [23] have been found to significantly improve lower limb strength, functional capacity, dynamic balance, and walking speed in menopausal women. These physiological improvements may be partly attributed to the intervention’s enhancement of neuroplasticity in brain regions associated with motor function, cognition, and somatosensory processing.

Specifically, dance, as an activity that integrates both physical exertion and cognitive challenges, helps to activate and restructure neural networks, thereby optimizing neural transmission and muscle coordination, ultimately improving motor performance [35,36,37]. Furthermore, the concept of “neuromuscular restructuring” in dance was first introduced in the scientific literature by Krasnow in 1997 [38], providing a theoretical framework for understanding this phenomenon. This concept emphasizes that during the execution of complex and coordinated movements, the nervous system continuously adjusts its connections to adapt to changes in the body and environment, thereby achieving functional optimization. Brodie and Lobel [39] also highlighted the crucial role of physical training in enhancing bodily coordination and neuromuscular control, further supporting the effectiveness of Zumba interventions in improving physiological function in menopausal women.

Most women experience a variety of uncomfortable symptoms during the menopausal transition, which are closely associated with the decline and fluctuation of estrogen levels. Estradiol, as the primary female gonadal hormone, plays a crucial role not only in maintaining reproductive function, but also in preserving cognition and memory; progesterone, another key female hormone and a biochemical precursor of estradiol, is indispensable for maintaining endocrine balance. Furthermore, although cholesterol is essential for cell membrane formation and normal cellular function, elevated serum cholesterol levels are closely linked to an increased risk of various diseases. The differential effects of the two major lipoproteins in the blood—low-density lipoprotein cholesterol (LDL-C) and high-density lipoprotein cholesterol (HDL-C)—on cardiovascular health are well recognized: excessively high LDL-C levels may lead to cholesterol deposition in the arterial walls, forming atherosclerotic plaques and thereby increasing the risk of heart disease and stroke, whereas higher HDL-C levels exert a protective effect. In addition, increased serum triglyceride levels are an important risk factor for multiple diseases, and are positively correlated with age-related cognitive decline [40]. Intervention studies on sports dance [21] have shown that, compared to control groups, menopausal women who participated in such interventions for more than one year experienced significant improvements in physical discomfort (e.g., menstrual irregularities, headaches, and fatigue) and demonstrated positive enhancements in mental health. Furthermore, a study on moderate-intensity sports dance interventions [22] revealed that such interventions not only significantly increased estradiol and progesterone levels, but also effectively reduced cholesterol, triglycerides, and low-density lipoprotein (LDL) cholesterol, while promoting an increase in high-density lipoprotein (HDL) cholesterol levels (*p* < 0.05). These changes suggest that dance interventions may exert protective effects at the physiological level by modulating hormone levels, thereby preventing chronic diseases associated with menopause. Regarding the underlying mechanisms of hormonal changes, relevant studies have provided compelling evidence [41,42]; notably, the research by Rodrigues-Krause et al. [43] further demonstrated that reductions in cholesterol levels yield significant overall health benefits.

In postmenopausal women, the gradual decline in estrogen levels is closely linked to the atrophy of the urogenital system. The reduction in estrogen is considered the primary trigger for the deterioration of urogenital function and its associated symptoms, such as urinary incontinence [44]. Studies on jazz dance interventions [24] have demonstrated that such interventions have significant effects in improving urogenital function in menopausal women (*p* = 0.001). Additionally, multiple studies have shown that moderate-to-moderately intense physical activity exerts positive effects on pelvic floor function, thereby reducing the incidence and risk of urinary incontinence [45], which is consistent with the findings of Magdalena Dąbrowska-Galas et al. [36]. Collectively, these results suggest that dance interventions may help to prevent and alleviate menopausal symptoms and related chronic conditions by restoring or maintaining estrogen levels and promoting hormonal balance [46,47], ultimately modulating endocrine function and the urogenital system.

Similarly, sports dance interventions [18] have demonstrated significant effects in improving bone mineral density (*p* < 0.05), rectus femoris muscle thickness (*p* < 0.05), muscle mass (*p* < 0.001), and fat mass (*p* < 0.001). These findings suggest that dance interventions may enhance body composition in late-perimenopausal and postmenopausal women through multiple mechanisms. Previous studies have found that late-perimenopausal and postmenopausal women typically experience declines in muscle mass [48,49,50,51], as well as loss of bone mass, which predisposes them to osteoporosis; conversely, high-intensity exercise interventions can help to maintain a more favorable muscle mass and fat distribution pattern and alleviate menopausal symptoms [36,52,53]. Specifically, dance training under load-bearing conditions may promote bone remodeling through mechanical stress, thereby increasing bone mineral density; at the same time, by enhancing muscle contraction and endurance, dance interventions may stimulate muscle synthesis, leading to improved muscle mass. These combined effects collectively reduce the risk of osteoporosis and sarcopenia.

Traditional Thai dance interventions [25] have demonstrated significant improvements in cardiopulmonary endurance and pulmonary function after six weeks of continuous intervention, highlighting their potential to enhance physical fitness. This finding has also been corroborated by the study conducted by Klonizakis et al. [54]. Specifically, the increased thoracic expansion observed post-intervention reflects improvements in diaphragmatic and intercostal muscle function, leading to enhanced respiratory efficiency and lung capacity [55]. Therefore, it is recommended that menopausal women actively engage in aerobic exercise to prevent health risks associated with the natural decline in pulmonary function.

Moreover, research on traditional Korean dance [20] reported significant changes in immunoglobulin levels and body temperature following the intervention, suggesting the potential role of dance in modulating immune function. Exercise is believed to activate the body’s immune response, thereby increasing serum immunoglobulin levels and improving serum ratios, ultimately enhancing overall immune function [56]. These results broaden the scope of dance as a health intervention tool.

Among the studies included in this review, only two [23,24] specifically evaluated menopausal symptoms. One study [24] utilized a standardized menopause rating scale, while the other study [23] selected participants with lower menopausal index scores based on specific parameters, but did not provide detailed quantitative indicators or score distribution data. Future research should incorporate menopause rating scales more extensively to conduct a more comprehensive and in-depth analysis of menopausal women.

Menopausal sleep disorders are a common and significant issue faced by many women. Existing studies have shown a high prevalence of sleep disorders among postmenopausal women. A meta-analysis conducted by Salari et al. [57] reported a prevalence rate of 51.6% (95% CI: 44.6–58.5%), while Gómez-Santos et al. [37] highlighted that postmenopausal women exhibit decreased circadian rhythm stability and an increased incidence of sleep disturbances. Additionally, irrespective of age, perimenopausal and postmenopausal women report significantly higher rates of sleep disorders compared to premenopausal women [58]. Menopausal sleep disorders may also serve as an independent risk factor associated with arterial stiffness, thereby increasing the incidence and mortality of cardiovascular diseases [59]. However, the studies included in this review did not contain data on sleep quality or circadian rhythm measurements. This limitation restricts the evaluation of the effectiveness of dance interventions in improving menopausal sleep disorders. Therefore, future research should give due attention to and assess sleep-related outcomes to determine whether dance interventions can improve sleep disturbances, ultimately providing a more comprehensive intervention strategy for menopausal women.

### 4.2. Strengths and Limitations

This study is the first to systematically review and meta-analyze the effects of dance interventions on health-related outcomes in perimenopausal, menopausal, and postmenopausal women, representing a novel contribution to the field. Subgroup analyses highlighted the differential impacts of weekly intervention frequency and total duration on depression and anxiety, providing evidence for optimizing intervention design and guiding future research. Additionally, this systematic analysis offers a reliable reference framework for promoting the use of dance interventions in menopausal health.

However, the study has several limitations. The number of included studies was small (only five were eligible for meta-analysis), limiting the statistical power of the analyses. Furthermore, high heterogeneity in depression, anxiety, somatization, and psychotic symptoms complicated the result interpretation. Due to limited sample sizes, some subgroup analyses were constrained, and the generalizability of the findings should be interpreted cautiously.

### 4.3. Future Directions

Despite the limited number of included studies, this research provides insights into potential moderating factors, and serves as a reference for future study designs. An ongoing study in Greece (Trial ID: NCT06260124) [60] is recruiting menopausal women to evaluate the acute effects of Greek traditional dance on health, physical performance, and muscle damage-related parameters. Such research holds promise for further validating the positive effects of dance interventions on menopausal women, encouraging more studies to explore their benefits for perimenopausal, menopausal, and postmenopausal populations.

## 5. Conclusions

This study included nine trials, and found that dance interventions had significant positive effects on health-related outcomes in perimenopausal, menopausal, and postmenopausal women, except for psychotic symptoms. However, given the relatively small sample size of the included studies, the findings should be interpreted with caution. Future research with robust study designs and high-quality methodologies is recommended to validate these results and provide stronger guidance for clinical practice.

To further validate and promote the effectiveness of dance interventions, future research is recommended to optimize study design by employing large-scale, high-quality experiments with sufficient sample sizes to enhance the robustness and external validity of the findings; moreover, incorporating comprehensive measures such as menopause symptom scales and sleep quality data into the assessments will ensure the comprehensiveness of the results.

## Figures and Tables

**Table 1:** A list of basic characteristics of the studies included in the meta-analysis.

Study ID and Year	Country/Type	Sample (EG/CG)	Average Age (EG/CG)	Type of Intervention		Intervention Cycle	Duration of Intervention (Hours)	Weekly Intervention Frequency	Total Duration of Intervention
				EG	CG				
Waer et al., 2024 [23]	Tunisia/RCT	EG: 16, CG (Pilates group): 16, CG (waiting group): 16	57 ± 2	Zumba	The Pilates group performed training/the control group had no program	12 weeks	Each session lasted 60 min	3 times a week	2160 min
Martins et al., 2023 [24]	Brazil/RCT	EG:23, CG:24	None	Jazz	No changes in lifestyle and physical exercise habits (no dancing of any kind)	16 weeks	Each class lasted 60 min	2 times a week	1920 min
Janyacharoen et al., 2015 [25]	Thailand/RCT	EG:31, CG:32	EG: 54.6 ± 6.4 CG: 53.7 ± 7.8	Thai Traditional Dance	No exercise program or general health guidance	6 weeks	60 min each time	3 times a week	1080 min
So et al., 2012 [17]	Korea/RCT	EG:14, CG:13	EG: 50.2 CG: 51.1	DMT	Non-intervention	15 weeks	90 min each time	1 time a week	1350 min
Zhao, 2006 [22]	China/RCT	EG:38, CG:22	47.4 ± 2.9	Sports Dance	Daily activities	6 months	Each session was more than 50 min	5–6 days a week	4800–7200 min
Kim et al., 2012 [18]	Korea/RCT	EG:10, CG:10	EG: 55.5 ± 3.5 CG: 57.0 ± 3.3	Sports Dance	None	8 weeks	60 min each time	3 times a week	2400 min
Kim, 2023 [20]	Korea/RCT	EG:8, CG:8	EG: 53 ± 3.9 CG: 54.0 ± 4.2	Korean Traditional Dance	None	12 weeks	60 min each time	3 times a week	2160 min
Kim, 2006 [19]	Korea/non-RCT	EG:35, CG:35	None	Sports Dance	None	12 weeks	50 min each time	3 times a week	1800 min
Pan, 2004 [21]	China/non-RCT	EG:41, CG:40	None	Sports Dance	Free time	6 months	60–90 min each time	4–6 times a week	2880–6480 min

## Data Availability

The data supporting the study’s findings are available from the first author upon reasonable request.

## Abbreviations

The following abbreviations are used in this manuscript:

HT	Hormone therapy
NHDT	Non-hormonal drug therapy
CHD	Coronary heart disease
SMD	Standardized Mean Difference
MD	Mean Difference
ROB2.0	Risk of Bias 2.0
ROBINS-I	Risk of Bias in Non-randomized Studies of Interventions
I^2^	I-Square
DMT	Dance movement therapy
CI	Confidence interval
P	P-value
LDL-C	Low-density lipoprotein cholesterol
HDL-C	High-density lipoprotein cholesterol
